# Nr3C1-Bhlhb2 Axis Dysregulation Is Involved in the Development of Attention Deficit Hyperactivity

**DOI:** 10.1007/s12035-015-9679-z

**Published:** 2016-01-28

**Authors:** Li Hui Wu, Wei Cheng, Mei Yu, Bao Mei He, Hui Sun, Qi Chen, Yi Wei Dong, Xiao Ting Shao, Qian Qian Cai, Min Peng, Xing Zhong Wu

**Affiliations:** 1 Department of Children’s Health Care, The Second Affiliated Hospital & Yu Ying Children’s Hospital, Wenzhou Medical University, Wenzhou, China; 2 Key Lab of Glycoconjugate Research, Ministry of Public Health, Department of Biochemistry and Molecular Biology, Shanghai Medical College, Fudan University, 138 Yi Xue Yuan Road, Shanghai, 200032 China; 3 Department of Clinic Medicine, Zhejiang Medical College, 481 Binwen Road, Binjiang College Zone, Hangzhou, 310053 China; 4 Department of Neonatology, Shanghai First Maternity and Infant Hospital, Tongji University School of Medicine, Shanghai, China

**Keywords:** miRNA, Glucocorticoid receptor, DEC1, ADHD

## Abstract

Attention deficit hyperactivity disorder (ADHD) is a child developmental and behavioral disorder which seriously hinders their education and development. To investigate the key regulators in the prefrontal cortex (PFC), the major affected areas of ADHD, microRNA (miR)-138,138*, 34c*, 296, and 494, were noted for their significant downregulation in ADHD model rats spontaneously hypertensive rats (SHRs) compared to Wistar Kyoto (WKY) rat control. Based on promoter sequence analysis and activity assay, glucocorticoid receptor (Nr3c1) was identified for the inhibition of the promoter activity of miR-138-1, 34c*, 296, and 494 genes and their transcription. In the PFC of ADHD model rats SHR, Nr3c1 expression was abnormally elevated and reversely correlated with the levels of miR-138-1, 34c, 296, and 494 expression. Luciferase report assays indicated that all miR-138, 138*, 34c*, 296, and 494 targeted the 3′ untranslated region of transcription factor Bhlhb2 (Bhlhe40) messenger RNA (mRNA) in common and ectopic expression of miR-138,138*, 34c*, 296, and 494 further suppressed the expression of Bhlhb2 gene. Consistently, Bhlhb2 expression was significantly higher in PFC of ADHD model SHR than control. Overexpressed Bhlhb2 in vitro significantly suppressed PC12 cell differentiation, and silence of Bhlhb2 enhanced the growth of neurite axon and dendrite. To observe the roles of Bhlhb2 further in vivo, Bhlhb2 was silenced in the PFC of nine SHR rats. Interestingly, knockdown of Bhlhb2 significantly improved the hyperactivity behaviors in SHRs compared to control. These findings show that Nr3c1-Bhlhb2 axis dysregulation was involved in the development of attention deficit and hyperactivity.

**Electronic supplementary material:**

The online version of this article (doi:10.1007/s12035-015-9679-z) contains supplementary material, which is available to authorized users.

## Introduction

Attention deficit hyperactivity disorder (ADHD) is a child behavioral and developmental disorder characterized by age-inappropriate inattention, impulsiveness, and hyperactivity. The disorder might persist to adult and hence seriously hinder their education and psychology during the development. Persistent deficits in attention are often linked with academic underachievement, underemployment, and interpersonal communicating problems [1]. Patients with ADHD often show substantial impairment in social functioning, academic attainment, and cognitive functioning [2] which are important for the development of children and their future career. ADHD is thus a developmental and behavioral disorder in childhood, which needs extensive study to understand its development with variable clinic outcomes and to improve the treatment response. Although recent advances in molecular genetics underlying ADHD, the heterogeneous symptoms of ADHD still cannot be explained only based on current understanding and the key molecules with dysregulation and malfunction in the brain and responsible for the attention deficit hyperactivity remains largely unknown. A study with new strategy forward to reveal the core mechanisms that underpin and robustly explain the variable symptoms of ADHD [3] is thus a public health priority. MicroRNAs (miRNAs) have been implicated in several neuronal processes, such as behaviors and biological rhythms. In the children and adolescents with ADHD, there are several circulating miRNAs which are differentially expressed compared to control healthy children [4]. The development of human brain especially PFC may be related with the gene expression controlled by miRNAs that are rich in the brain [5]. Our previous study showed that miRNA let-7d was abnormally expressed in the prefrontal cortex (PFC), the major affected brain area in ADHD, in spontaneously hypertensive rats (SHRs), a well-established animal model of ADHD with similar therapy response to human [6], and the abnormal expression of let-7d was associated with the regulation of tyrosine hydroxylase [7]. In the central nervous system (CNS), miRNAs are particularly abundant and are very important in regulation of neuronal activity, inking them to nerve diseases. Brain-derived neurotrophic factor (BDNF), on the other hand, modulates the strength of existing synaptic connections and helps form new synaptic contacts [8] and is thought to be related with the susceptibility to ADHD [9]. The expression of BDNF is, however, controlled by basic helix-loop-helix transcription factor Bhlhb2 (Bhlhe40), which is highly expressed in the brain. Bhlhb2 binds to class B E-box sites on its gene promoter DNA, either as heterodimers or homodimers, and regulates gene expression [10]. However, the regulation of Bhlhb2 expression and its roles are not well understood, especially in the brain, although abnormal regulation of BDNF is important in neuronal activity and locomotor control. Based on our previous observation, in this study, we identified Bhlhb2 as the critical player in the gene expression network regulated by miRNAs through analyzing and screening the intercross of differential miRNA, messenger RNA (mRNA), and protein expression profiles in the PFC of ADHD model rats.

## Materials and Methods

### Animals

Six-week-old juvenile male SHR and age- and sex-matched control Wistar Kyoto (WKY) rats weighing from 120 to 150 g were obtained from Shanghai Slac Laboratory Animal CO. LTD (Shanghai, China). SHR has been a well-established animal model for ADHD (at this age, SHRs do not develop hypertension) [6, 11] when WKYs have been used as the closest genetic control for the SHR. All the experiments were performed according to the Guidelines for the Care and Use of Laboratory Animals and approved by the Animal Ethics Committee of Fudan University Shanghai Medical College with the permit number of 20120302-003.

### ADHD-Related Behavioral Assessments

#### The Open-Field Test

General activity levels and anxiety were measured with the open-field test [7]. Briefly, the open field (90 cm × 90 cm × 50 cm) was divided into 81 squares, each 10 × 10 cm and illuminated by a white, cold 4-W lamp 100 cm above the floor. Rats (*n* = 6) were placed in the center and allowed to move freely for 60 min, from 11:00 a.m. to 12:00 noon. The behavior was recorded by a video camera. Ambulation and rearing were defined as the number of squares crossed with all four paws and the number of times the animals stood upright on their hind limbs, respectively.

#### The Làt Maze Test

Locomotor activity levels and non-selective attention were analyzed in Làt maze test according to previous report [11]. A 60 × 60 × 40-cm black cage contained a 30 × 30 × 40-cm transparent plastic smaller box in the middle to result a 60 × 15 × 40-cm corridor. Both six SHR and six WKY rats were allowed to explore the corridor around the periphery of cage. The rat’s movements were tracked over 30 min from 4:30 a.m. to 5:00 a.m. by a video camera. The frequencies of rearings (the number of times the rats stood upright on their hind limbs) and corner crossings (the number of times the rats passed by the corner) were recorded.

#### The Step-Down Test

The step-down test according to Rezayof [12] was conducted in a 30 × 30 × 40-cm-high box, the floor of which consisted of parallel stainless steel bars, 0.3-cm diameter spaced 1 cm apart. A 4 × 4 × 4-cm wood block was placed in the corner on the floor. The animal was placed on the grid floor for 3 min, and it received a continuous electrical shock (50 Hz, 0.3 mA, 48 V) for 5 min through the grid floor. When shocked, the number of times that the rat jumped off the block in the 5 min was recorded. Twenty-four hours after the training, the animal was placed on the block and the interval from being on the block to jumping off by placing four paws on the grid floor was measured as latency time. The numbers of jumps onto the platform were measured as a learning score to reflect memory activity. The animals included in this observation consisted of six SHR and six WKY rats.

### Bioinformatics

The target mRNA of all miRNA was searched through the database of microCosm, Pictar, and TargetScan based on the data of microarray in our previous study [7]. The target mRNAs and miRNAs were negatively associated to construct a miRNA–mRNA network. Transcription factor binding consensus was then analyzed in the upstream sequence of all miRNA precursor genes and the differentially expressed protein-coding genes based on previous complementary DNA (cDNA) array [13] and proteomics profiles [14]. The richest transcription factors were then identified after integration analysis of either differentially expressed miRNA or protein coding genes.

### Vector Construction

Rat Nr3c1 and Bhlhb2 full-length cDNAs (NM_012576.2; NM_053328.1) were obtained by reverse transcription–polymerase chain reaction (RT–PCR) from rat brain RNA. The primers for Nr3c1 consisted of 5′AATGGACTCCAAAGAATCCT3′ (sense 1) and 5′CATGCCTCCACGTAACTGT3′ (antisense 1); 5′ATGGACTCCAAAGA3′ (sense 2) and 5′TTTTTGATGAAACA3′ (antisense 2). The amplified cDNA was cloned into pcDNA3.1B at the sites of *Bam H1* and *Xba I*. The primers for Bhlhb2 consisted of 5′ATGGAGCGGATCCCC3′ and 5′GTTTAGTCTTTGGTTTCTAAGTTT3′. The amplified cDNA was cloned into pcDNA3.1B at the sites of *EcoR I* and *Xba I*. Neurofibromin plasmid was kindly provided by professor Shibahara [15].

The precursors of miR-138, miR-138*, miR-296, miR-34c*, and miR-494 were obtained by PCR from rat genomic DNA and cloned into *Hpa I* and *Xho I* sites of lentivirus pLL3.7. The upstream sequence of Bhlhb2 (2.5 kb) and the pre-miRNA genes (3 kb) were amplified by PCR and cloned into pGL3-Basic firefly luciferase report plasmid at the *Kpn I* and *Xho I*, or *Mlu I* and *Hind III* sites. The 3′ untranslated region sequences of Bhlhb2 mRNA were cloned into psiCHECK-2 dual-luciferase report plasmid. All constructs were confirmed by DNA sequence analysis.

### RT-PCR

cDNA was obtained by specific reverse transcription. PCR was performed with a cycler (BS-196, Dongsheng, Beijing, China), and the band density after electrophoresis was analyzed for semiquantification. Real-time PCR was performed with an iQ5 cycler (Bio-Rad, Hercules, CA, USA) and quantitative analysis was achieved by delta Ct method (specific primers for miRNAs are listed in Supplemental Table 1). All reactions were run in triplicate. The data were obtained by normalizing to the interior control [RNU6 for miRNA, glyceraldehyde 3-phosphate dehydrogenase (GAPDH) for mRNA] and shown as the relative level to the control.

### Immunofluorescence

Immunofluorescence was performed as described [7]. The brain sections were permeabilized, blocked by goat serum, and then incubated with the primary antibodies against Nr3c1, Bhlhb2, and BDNF (Santa Cruz, CA, USA), respectively. The secondary antibody was rhodamine-conjugated goat anti-rabbit antibody. The nuclei were stained with 4′,6-diamidino-2-phenylindole (DAPI). Sections were then observed under an Olympus fluorescence microscope. The relative fluorescent intensities were semiquantified with the ImageJ software package. At least 200 cells were analyzed in each group, and data were shown as means + SD.

### Luciferase Assay

The cotransfection for luciferase assay was based on our previous report [7] and performed in highly differentiated rat kidney pheochromocytoma PC12 (PC12H) cells, human embryonic kidney HEK-293T cells or rat hepatoma CBRH-7919 cells. Luciferase activity was analyzed with the Luciferase Reporter Assay kit (Promega, USA) according to the manufacturer’s instructions. The data of relative luciferase activities were normalized to the control. At least three independent assays were performed.

### miRNA Mimics

Oligoribonucleotide was synthesized by GenePharma Company (Shanghai, China) according to mature miRNA sequence and used as the miRNA mimics. The negative control (NC) was also provided by the manufacturer.

### Western Analysis

The cell and tissue lysates were loaded onto and resolved by 10 % sodium dodecyl sulfate–polyacrylamide gel electrophoresis (SDS-PAGE). After electrophoresis, the proteins were blotted to a polyvinylidene difluoride membrane. After blocking with 5 % milk protein, the membrane was incubated with primary antibody and horseradish peroxidase-conjugated anti-rabbit or anti-mouse antibody. The band was visualized with enhanced chemiluminescence kit according to the manufacturer instruction, and the image was captured by an EMCCD Imaging System (Tenon, Shanghai, China). The protein levels were determined with Lowry method.

For immunoprecipitation (IP) assay, tissues were lysed in cold IP lysis buffer (50 mM Tris–HCl, pH 7.5, 150 mM NaCl, 1 % NP-40, 2 mM EDTA, 1 mM phenylmethylsulfonyl fluoride, 5 mM NaVO4,10 μg/mL leupeptin, and 10 μg/mL aprotinin) and treated with ultrasonication on ice for 20 min. The lysates were centrifuged (12,000 rpm, 20 min, 4 °C), and the supernatant was precleared with normal rabbit IgG and protein A/G-agarose beads. Then, the lysate was incubated with Nr3c1 or Bhlhb2 primary antibody and protein A/G-agarose beads and rotated overnight at 4 °C. After washing with IP buffer, the beads were added with loading buffer and boiled to release the bound proteins for Western analysis.

### PFC Microinjection

The SHR rats were anesthetized and then placed on a standard stereotaxic device with the skull flat. After the scalp was incised, a bilateral guide was placed through an indwelling stainless steel cannula (interior diameter, 0.33 mm; external diameter 0.63 mm) to reach the right PFC with the co-ordinates +3.0 A, −0.8 L, and −0.2 V measured from the dura. Then, 25 pmol siRNA oligonucleotide: GCACGUGAAAGCAUUGACAdTdT, UGUCAAUGCUUUCACGUGCdTdT with cholesterol and 2′-*O*-methylated modification (Biomics Biotechnologies, Nantong, China) were injected into each of seven SHRs through an inner cannula (interior diameter, 0.08 mm; external diameter, 0.3 mm, which protruded from the guide cannulae by 1.6 mm) by a microprocessor pump at a rate of 250 nL/min for 2 min. The injection needle was left in the tissue for another 2 min after the infusion until diffusion was complete. Seven SHRs were injected with the same amount of scramble oligonucleotide as the NC. Coordinates for rat brains were based on the atlas by Paxinos and Watson [16]. The behavioral changes of all rats were closely observed after the operation except one rat died during the operation.

### Differentiation Morphology Analysis

PC12 cells were plated onto 60-mm tissue culture plates at a relatively low level of density. Two or three hours after plating, the medium was replaced with fresh medium containing 1 % fetal bovine serum and 10 ng/mL nerve growth factor (NGF) for 24–36 h. The cells were fed on days 2, 4, and 6 by the addition of fresh medium containing 10 ng/mL NGF. After treatments, neurite outgrowth was observed and the cells were photographed on days 0, 2, 4, and 6. Five fields were randomly selected and observed under a microscope. The cells bearing neurite outgrowth longer than 10 μm and more than 3 were counted as one with branching. The percentage of the cells with branching was calculated over total cells observed. The neurite length was measured by analyzing 150 cells from more than three randomly selected fields in each assay. Experiments were repeated at least three times independently.

### Corticosterone and Cortisol Measurements

Corticosterone in plasma samples collected from 12 SHR and 9 WKY rats at 8:30 a.m. was measured by ELISA assays according to the manufacturer’s instructions (Elabscience, Wuhang, China). Cortisol concentrations were measured from 30 Chinese Han boys between 6 and 10 years old with a diagnosis of ADHD [17] who were recruited between September 2010 and October 2012 from the psychiatric outpatient’s clinic at Yu Ying Children’s Hospital. Informed consent was obtained from their parents.

### Statistical Methods

All experiments were conducted independently at least three times. Data are expressed as the means and standard errors. Group means were compared with Student’s *t* test for two groups and with one-way ANOVA for three or more groups. Post hoc analysis was also performed. All data met the assumptions of the tests used to analyze them. Alpha was set at 0.05, and all tests were two-tailed.

## Results

### Behavioral Assessment of the Animals

**Fig. 1:**
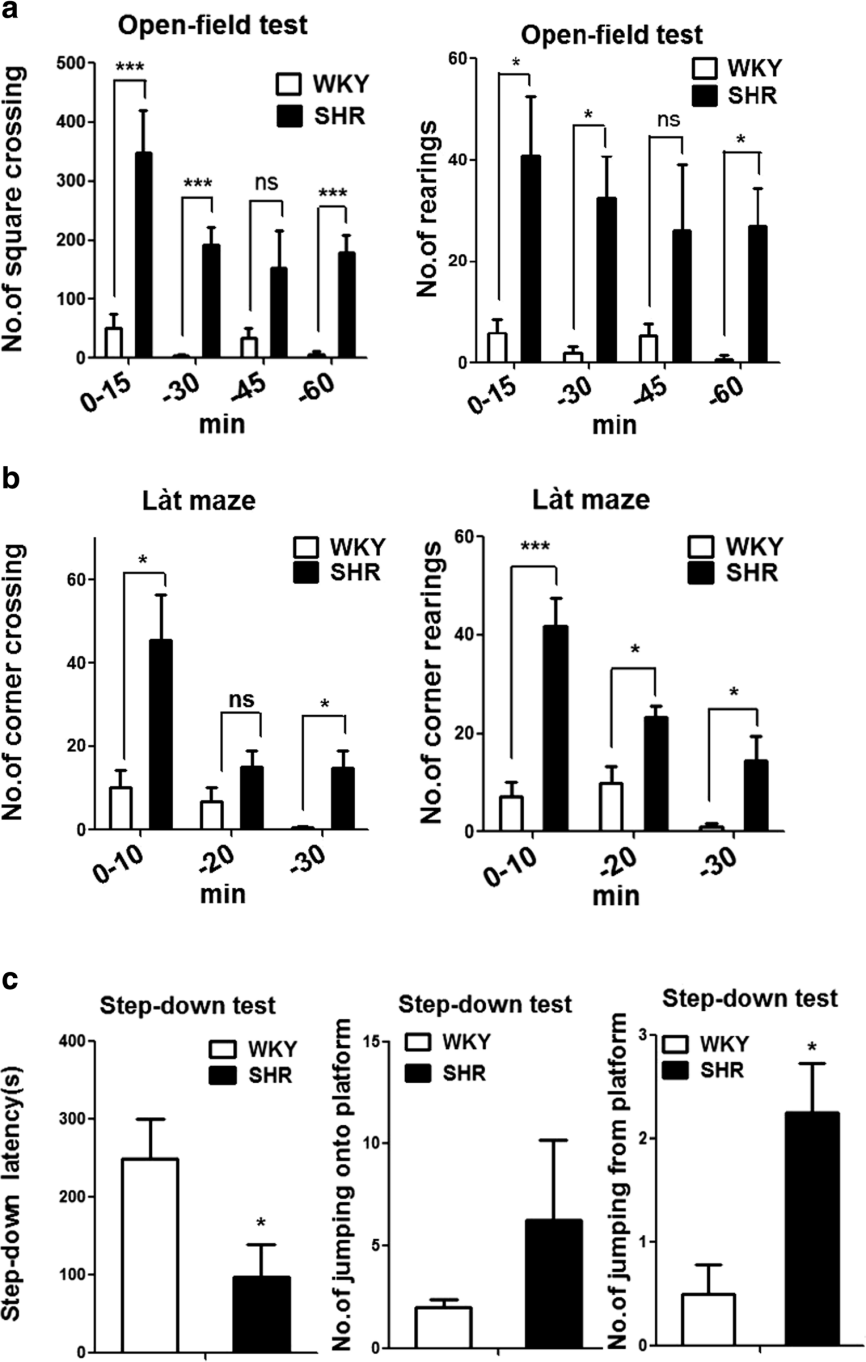
Outcomes of the behavioral tests. **a** Square crossing and rearing analysis in open-field test. **b** Behavior analysis in Làt maze test. **c** Step-down analysis. Data are the average of four independent experiments with standard deviation. *Ns* not significant at the 005 level. ***P < 0.001, **P < 0.01, *P < 0.05

In the open-field test, SHRs were more active than the WKY rats. The total number of square crossings and the number of rearings were significantly higher in SHRs than in WKY rats (Fig. 1a). In the Làt maze test, the numbers of corner crossings and rearings were analyzed in every 10 min and were significantly higher in SHR (Fig. 1b), especially in 10-min group. Mean learning scores from the step-down test did not differ significantly between groups, but the latency time was significantly shorter in SHRs (Fig. 1c), and the number of wrong jumps from the platform onto the grid floor was significantly higher in SHRs. These results validated the SHR as ADHD model, which was then used in the following experiments.

### Screening Analysis of miRNAs and Related Gene Network

**Fig. 2:**
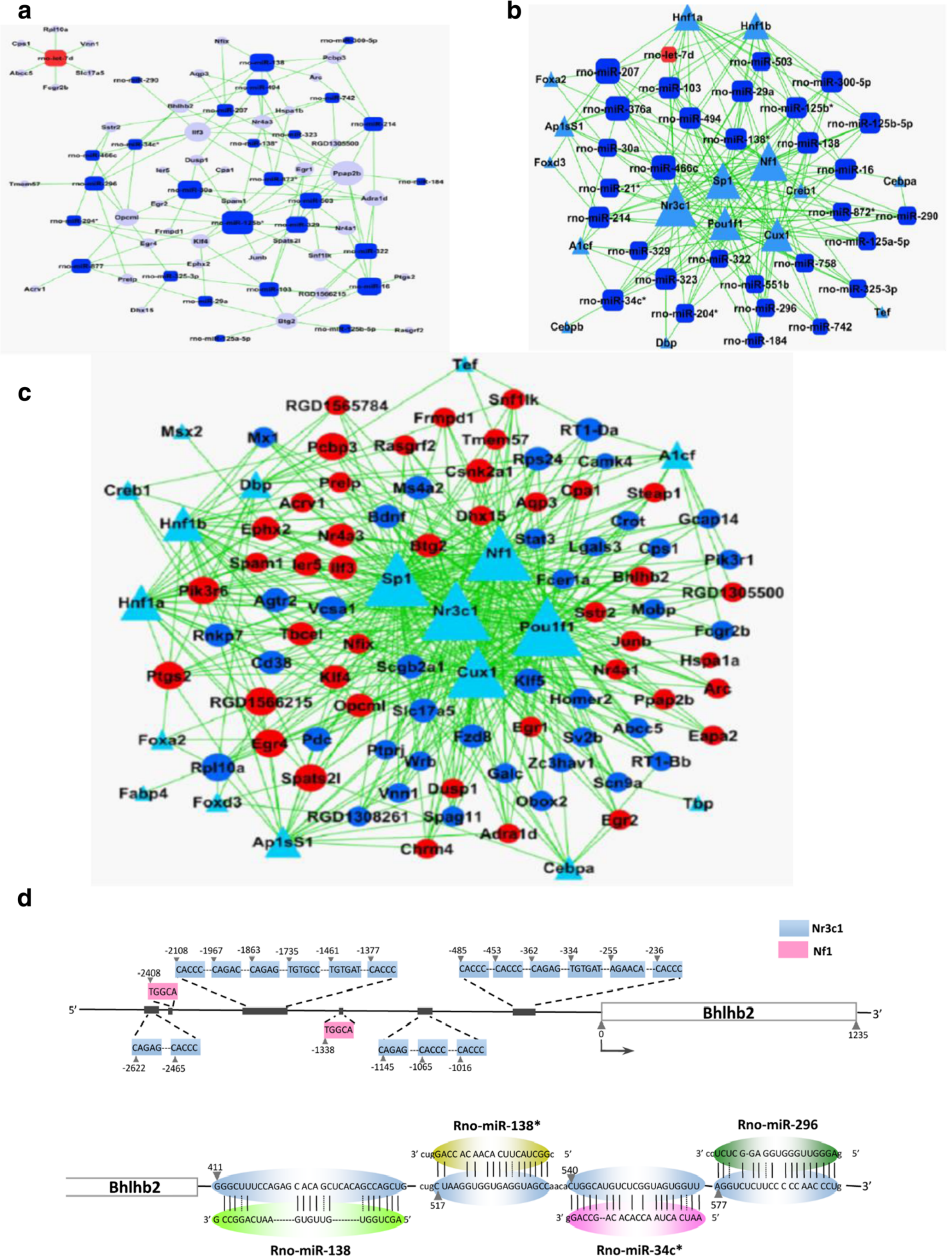

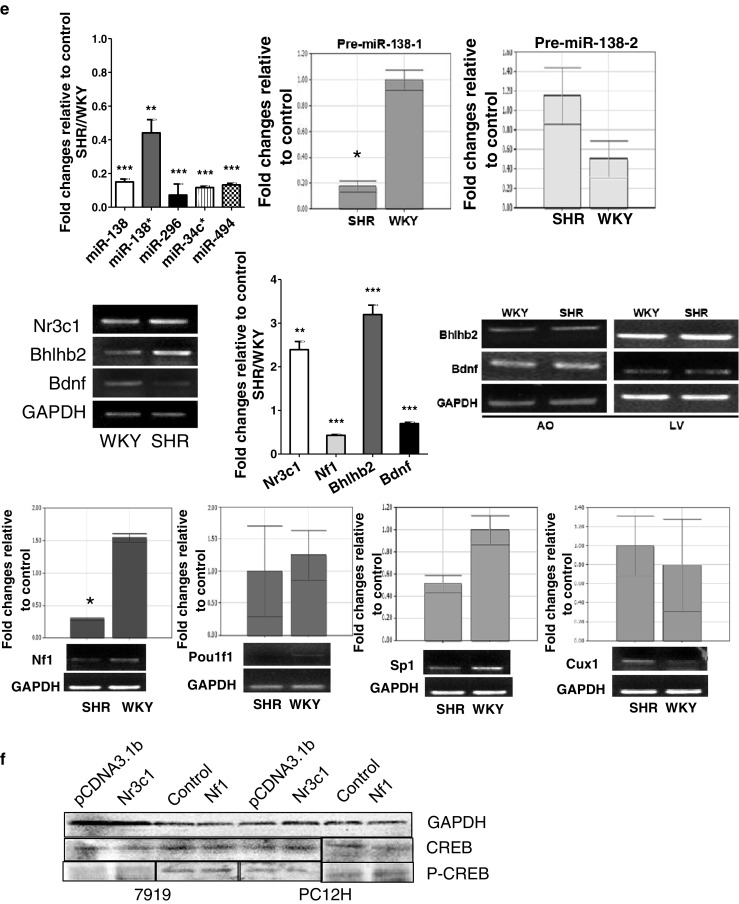
Transcription factor network analysis and confirmation. **a** The miRNA and their related mRNA network based on the microarray results of the prefrontal cortex (PFC) in SHR and WKY rats. *Red* indicates increased miRNA; *blue* indicates decreased miRNA in SHR; the *gray* indicates the putative target mRNAs. **b** Transcription factor and miRNA gene network. *Triangles* represent a transcription factor, and the *area of the triangle* indicates the degree that the transcription factor binds to the putative promoters of miRNA genes. **c** The network of transcription factors and the related proteins that were differentially expressed in SHR. *Red spheres* indicate the increased proteins, and *blue spheres* indicate decreased proteins in SHRs compared to WKY rats. The *area of the triangle* (transcription factors) represents their degree in regulation of the protein expression. **d** The upstream sequence of Bhlhb2 gene contains Nr3c1 binding consensus. The 3′ untranslated region of Bhlhb2 mRNA can be targeted by miR-138, 138*, 34c*, and 296. **e** Expression analysis of miRNAs and Nr3c1. The relative expression of the miRNAs and miRNA precursors (Pre-miRNA-138-1, Pre-miRNA-138-2) in the PFC of SHR and WKY rats was measured by real-time PCR (*top*). The expressions of Nr3c1, Bhlhb2, and BDNF were detected in the PFC by RT-PCR and real-time PCR (*left* and *central middle panel*). Bhlhb2 and BDNF in the aorta (AO) and liver (LV) of both SHR and WKY rats were detected by semiquantitative RT-PCR (*right middle panel*). The expressions of Nf1, Pou1f1, Sp1, and Cux1 were also detected in the PFC by real-time PCR (*bottom panel upper*) and semiquantitative RT-PCR *(bottom panel lower*). **f** The expression and phosphorylation of transcription factor CREB were observed in PC12H and CBRH-7919 cells transfected with Nr3c1 or Nf1 construct and its control vector pcDNA3.1b or PRC. Data are representative of three independent experiments. ***P < 0.001, **P < 0.01, *P < 0.05

In our previous study [7], microarray hybridization preliminarily identified 32 miRNAs as the differential expression between SHRs and WKY rats. Among these miRNAs, one miRNA was upregulated, and 31 miRNAs (Fig. 2a) were downregulated, respectively, in the PFC of SHRs compared to WKY rats. We further identified the miRNA-associated genes through integration analysis of mRNAs that were all differentially expressed in the PFC of SHRs according to the results of cDNA array [13]. We then obtained a miRNA–mRNA network (Fig. 2a). Further analysis of the upstream sequence of the miRNA precursor genes and the miRNA-associated genes revealed that glucocorticoid receptor Nr3c1 and transcription factors (Pou1f1, Sp1, Nf1, and CUX1) had their highest degree in binding of the putative regulation elements (Fig. 2b). Using the differential proteomic profiles in the PFC [14], the transcription factors that could potentially regulate the genes encoding the differentially expressed proteins were identified (Fig. 2c). Among these factors, Nr3c1, Pou1f1, Sp1, Nf1, and CUX1 had the highest degree in having the binding consensus in the upstream of the genes encoding the proteins differentially expressed in SHRs. All these factors overlapped with the putative regulators for the miRNA genes. The common potential target of miR-138*, miR-138, miR-296, miR-34C*, and miR-494 focused on the mRNA of Bhlhb2 (Fig. 2d), which encodes a basic helix-loop-helix domain-containing protein. Nr3c1 and transcription factors (Pou1f1, Sp1, Nf1, and CUX1) all have putative binding sites upstream of either the miR-138*, miR-138, miR-296, miR-34C*, or miR-494 genes or the Bhlhb2 gene (Supplemental Table 2, Fig. 2d). We then tested this regulatory network in the SHR brain.

### miR-138, 138*, 296, 34C*, and 494 in PFC

Expression levels of the miRNAs were confirmed with measurements in the PFC by qPCR. Compared to their expression in WKY rats, miR-138, miR-138*, miR-296, miR-34C*, and miR-494 expressions were significantly downregulated (Fig. 2e) in SHRs, results consistent with those of the miRNA microarray. Mature miR-138 is encoded by both miR-138-1 and miR-138-2 genes, whereas miR-138* is only encoded by the miR-138-1 gene. Further analysis of the precursors of miR-138 with qPCR showed that miR-138-1 was significantly downregulated in SHRs, but miR-138-2 not, suggesting that mature miR-138 was mostly from the miR-138-1 gene (Fig. 2e).

### Nr3c1 Expression Is Elevated in SHR

**Fig. 3:**
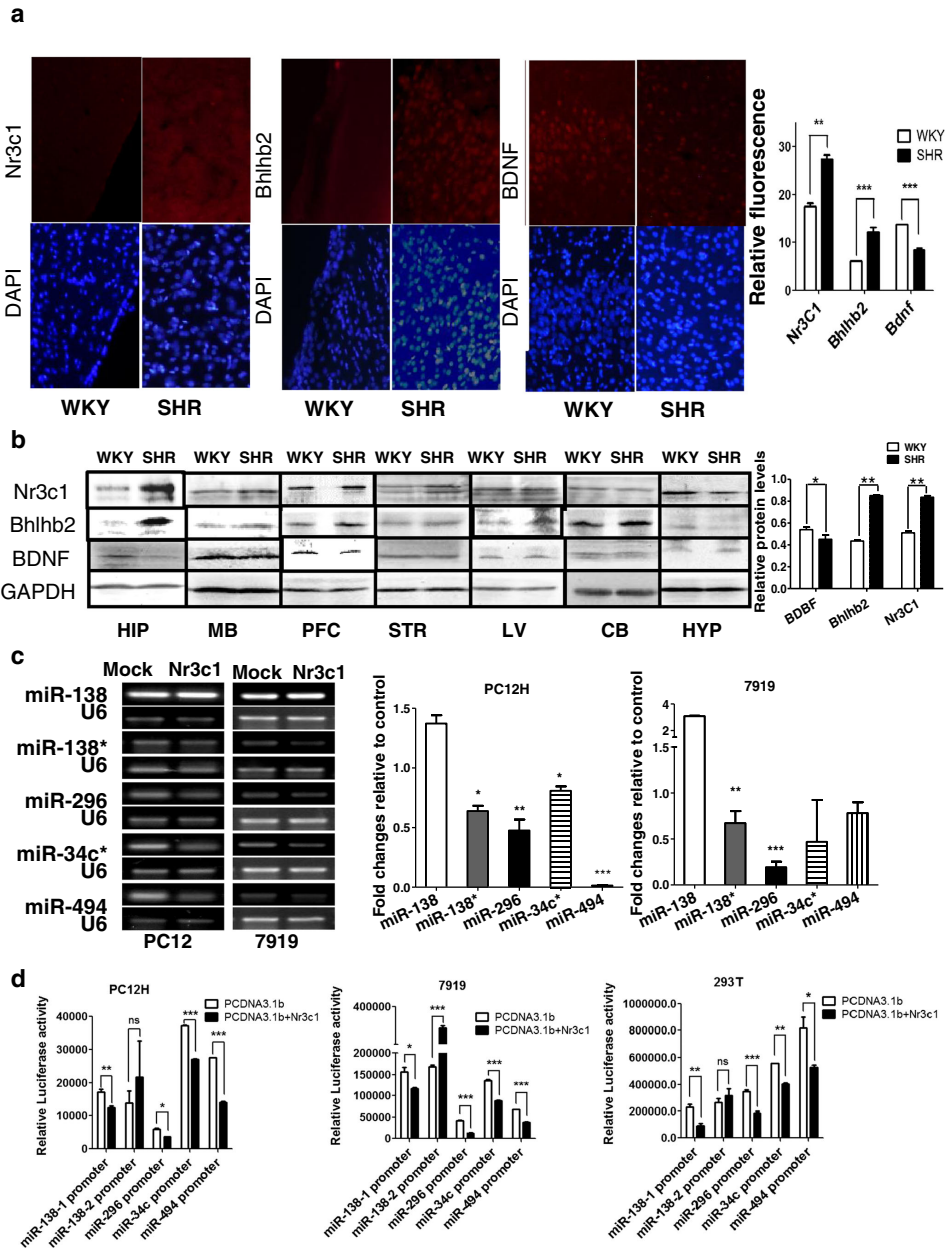
The miRNA gene regulation. **a** Immunofluorescent staining of Nr3c1, Bhlhb2, and BDNF in the PFC of SHRs vs WKY rats and measured by the signal intensity analysis (*right*). **b** Protein levels of Nr3c1, Bhlhb2, and BDNF were analyzed by Western analysis in the hippocampus (HIP), midbrain (MB), prefrontal cortex (PFC), striatum (STR), liver (LV), cerebellum(CB), and hypothalamus (HYP). Quantitative analysis in PFC is on the *right*. **c** miRNA-138, 138*, 296, 34c*, and 494 were detected by either semiquantitative RT-PCR (*left*) or quantitative PCR (*middle* and *right*) in PC12H and CBRH-7919 cells 24 h after transfection with Nr3c1 construct. **d** Reporter assay for the promoter of miRNA-138-1, 138-2, 296, 34c*, and 494 genes. The reporter activities were analyzed in Nr3c1-transfected cells and compared to those in the control vector. Data are representative of three independent experiments. ***P < 0.001, **P < 0.01, *P < 0.05

Bioinformatics analysis predicted that the upstream sequences of miR-138-1, miR-138-2, miR-296, miR-34c*, and miR-494 genes would have putative binding sites of Nr3c1 and transcription factors including CUX1, Pou1f1, Nf1, and Sp1 (Fig. 2b). Thus, we measured the expression levels of those factors in the PFC of both SHRs and WKY rats with qRT-PCR and semiquantitative RT-PCR. Nr3c1 expression was significantly higher in the SHRs (Fig. 2e); CUX1 expression increased slightly (Fig. 2e). The expressions of Nf1 and Sp1 were, however, downregulated, and Pou1f1 did not change significantly (Fig. 2e). cAMP response element binding protein (CREB) also did not change significantly (Fig. 2f). Nr3c1 was highly expressed in the PFC, hippocampus, midbrain, and striatum, but not in the hypothalamus (Fig. 3a, b). These suggested that there was a strong reverse correlation between the expressions of Nr3c1 and the miRNA (miR-138, miR-138*, miR-296, miR-34C*, and miR-494) and that Nr3c1 might be the main molecule responsible for the regulation.

### Nr3c1 Suppresses miR-296, 34c*, 494, and 138* Expressions

To investigate the regulatory effect of the elevated Nr3c1 in the brain on the miRNAs, we transfected PC12H cells and CBRH-7919 cells with Nr3c1 construct and measured the expression level of these miRNAs. The levels of all miR-296, 34c* 494, and 138*, except miR-138, were consistently suppressed in Nr3c1 transfectants in either RT-PCR or qRT-PCR measurements (Fig. 3c). To observe the direct regulatory effect of Nr3c1 on these miRNA genes, we constructed the reporter plasmid containing their promoters and cotransfected them into PC12H, HEK-293T, and CBRH-7919 cells with Nr3c1 expression plasmid. Nr3c1 significantly suppressed the promoter activities of all miR-296, 34c*, 494, and 138-1 genes, but not of the miR-138-2 gene (Fig. 3d).

### miR-296, 34c*, 494, 138, and 138* Target Bhlhb2

**Fig. 4:**
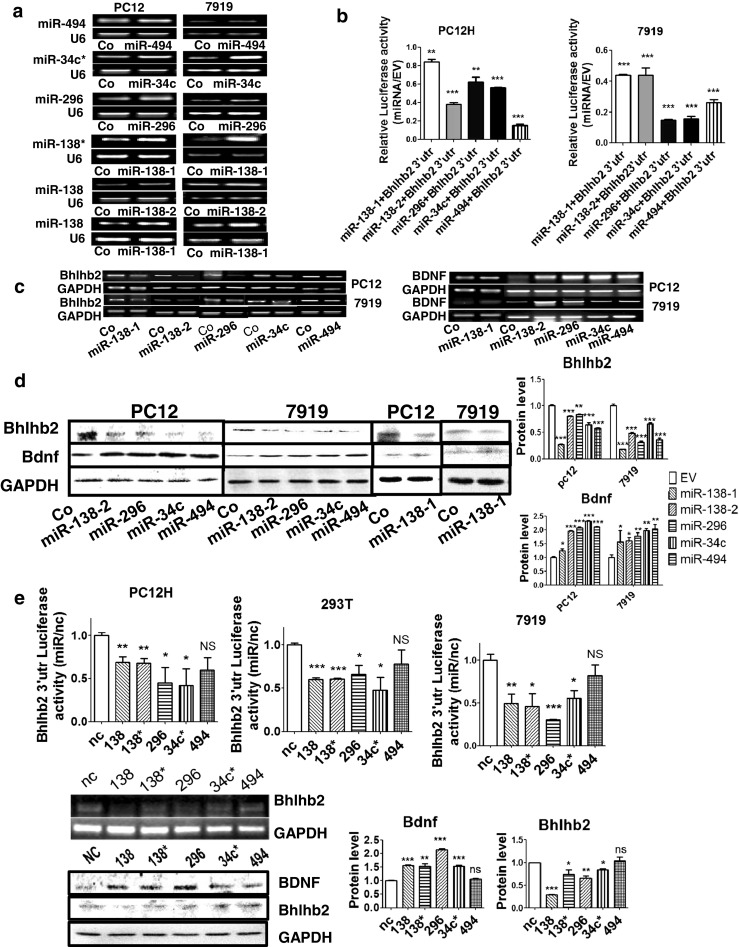
miRNA regulation of Bhlhb2 and BDNF. **a** The overexpression of miR-494, 34c*, 296, 138*, and 138 was validated by RT-PCR in PC12 and CBRH-7919 cells 24 h after transfection of the miRNA precursors. A lentivirus vector pLL3.7 containing unrelated sequence was used as a control. **b** Relative luciferase activities of Bhlhb2-3′UTR report plasmids were measured in PC12H and CBRH-7919 cells 36 h after cotransfection with miRNA expression vectors. **c** The expression levels of Bhlhb2 (*left*) and BDNF (r*ight*) were measured by RT-PCR 24 h after transfection with the indicated miRNA expression vector. **d** The protein levels of Bhlhb2 and BDNF were measured in both cell types after ectopic expression of the miRNA and quantified via densitometry (*right*). **e** The luciferase activities of the reporter encoding Bhlhb2-3′UTR were measured in three types of cells after the transfection of miRNA mimics (*top*). The levels of Bhlhb2 mRNA (middle) measured in PC12H cells after the transfection of miRNA mimics. Protein levels of Bhlhb2 and BDNF (*bottom*) measured by Western blot analysis in PC12H cells and measured with densitometry on the *right. NC* negative control. Data are representative of three independent experiments. ***P < 0.001, **P < 0.01, *P < 0.05

To investigate the roles of the miRNAs that were screened out in the SHR brain, we next analyzed the potential target of them and interestingly found that the 3′untranslated region of Bhlhb2 mRNA contained the target sequence of all miR-494, 34c*, 296, 138*, and 138 (Fig. 2d). Therefore, the lentivirus pLL3.7 vectors containing miRNA precursors were transfected into both PC12H and CBRH-7919 cells and the overexpression of miR-494, 34c*, 296, 138*, and 138 was confirmed (Fig. 4a). Then, the luciferase reporter, psi-Check 2.0 containing Bhlhb2-3′UTR, was cotransfected with either the miRNA precursor or a control vector, pLL3.7 (Fig. 4b), indicating that all the miRNAs transfected significantly suppressed the report luciferase activities in both cells.

After transfection with a miRNA-138-1, 138-2, 296, 34c*, or 494 precursor, Bhlhb2 mRNA (Fig. 4c) and protein (Fig. 4d) expressions were consistently suppressed. In contrast, BDNF expression was greatly elevated in both PC12H and CBRH-7919 cells (Fig. 4c, d), suggesting that these miRNAs targeted Bhlhb2 mRNA. To further confirm the action of the miRNAs, we employed synthetic miRNA mimics to transfect cells and observed their direct effects on Bhlhb2-3′UTR reporter activity. All the miRNAs except miR-494 significantly inhibited the reporter activity (Fig. 4e). The miRNA mimics also inhibited the mRNA and protein levels of Bhlhb2 (Fig. 4f) but enhanced BDNF expression. Among them, miR-138 and 296 inhibited Bhlhb2 the most and miR-494 the least.

### Nr3c1 Enhances Bhlhb2 Expression

**Fig. 5:**
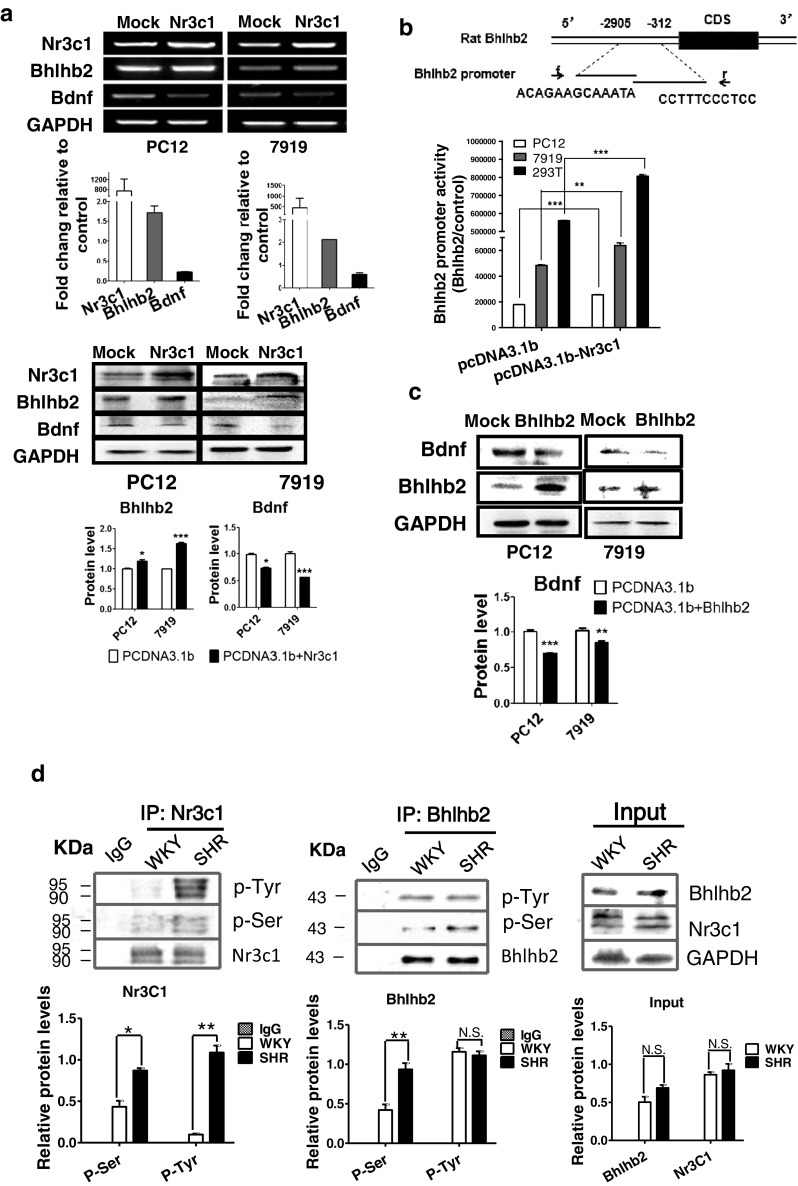
Nr3c1 regulation of Bhlhb2. **a** Nr3c1, Bhlhb2, and BDNF mRNA levels were detected by RT-PCR (*top*) and real-time PCR (*middle*) in two cell types, 24 h after transfection with Nr3c1 vector. Nr3c1, Bhlhb2, and BDNF protein levels were measured by Western analysis (*lower*) 48 h after transfection and measured with densitometry analysis (*bottom*). **b** Luciferase activities of Bhlhb2 promoter reporter plasmid were measured in PC12H, CBRH-7919, and HEK293T cells 36 h after cotransfection with Bhlhb2 promoter and Nr3c1 or Nf1 vectors. **c** BDNF and Bhlhb2 were measured by Western blot analysis and measured with densitometry in PC12H cells and CBRH-7919 cells transfected with either Bhlhb2 or Nf1 plasmid. **d** Phosphorylated Nr3c1 and Bhlhb2 were measured by coimmunoprecipitation with Nr3c1 or Bhlhb2 antibody. The precipitation was detected by the antibodies against phospho-tyrosine (p-Tyr) or phosphor-serine (p-Ser), and the quantitative analysis is in the *lower panel*

The miR-138, 296, 138*, 34c*, and 494 were downregulated in SHRs, a finding consistent with the high expression of Nr3C1 and Bhlhb2. Apart from the PFC, Nr3C1 and Bhlhb2 were also highly expressed in the hippocampus, midbrain, and striatum, but not in the hypothalamus or cerebellum (Fig. 3a, b). In all these sites, the expression of Nr3c1 was associated with that of Bhlhb2 (Fig. 3b). Importantly, overexpression of Nr3c1 (Fig. 5a) significantly stimulated Bhlhb2 expression in both PC12H and CBRH-7919 cells, as measured by both qRT-PCR and Western analysis (Fig. 5a). Nr3c1 also significantly stimulated the Bhlhb2 promoter report luciferase activities in three different cell types (Fig. 5b).

To further verify that Nr3c1 was an important regulator of the Bhlhb2 gene, we investigated the expression of BDNF, the downstream effecter of Bhlhb2 (Fig. 5c). We found that BDNF was suppressed after Nr3c1 was overexpressed in both PC12H and CBRH-7919 cells (Fig. 5a). We further investigated the phosphorylation of the transcription factors in the brain. The phosphorylation levels of Bhlhb2 protein in the PFC of SHR were almost similar to this in WKY, but Nr3c1 tyrosine phosphorylation was significantly strengthened in the PFC of SHR (Fig. 5d).

### Bhlhb2 Suppresses BDNF Expression

In cells transfected with the Bhlhb2 expression plasmid, the protein overexpression of Bhlhb2 was confirmed (Fig. 5c lower), and BDNF expression was significantly reduced in both PC12H and CBRH-7919 cells. The negative relationship between Bhlhb2 and BDNF expressions was also seen in the PFC (Fig. 3b). Bhlhb2 mRNA and protein levels were significantly higher in the PFC of SHRs than in that of the WKY rats (Figs. 2e and 3a, b), but BDNF expression in the PFC of SHRs was downregulated, which is related to recognition behaviors [18]. However, neither BDNF nor Bhlhb2 mRNA expression levels in the aorta or liver differed significantly between SHRs and WKY rats (Fig. 2e). Therefore, the regulation of Bhlhb2 and BDNF expression in SHRs was not systemic, but region-specific.

### Knockdown of Bhlhb2 Reduces Hyperactivity in SHR

**Fig. 6:**
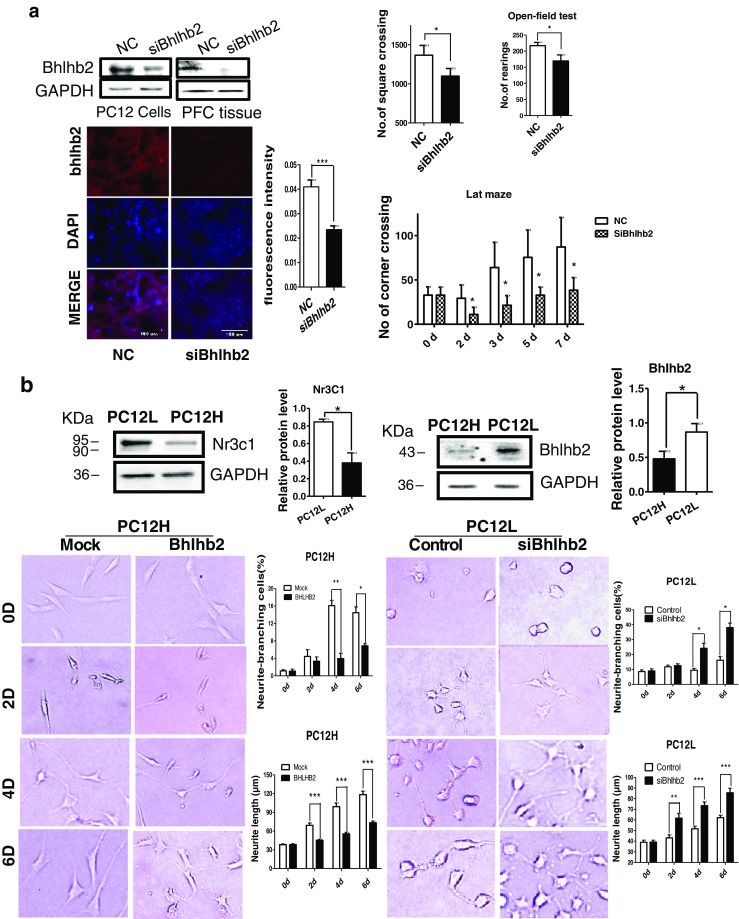

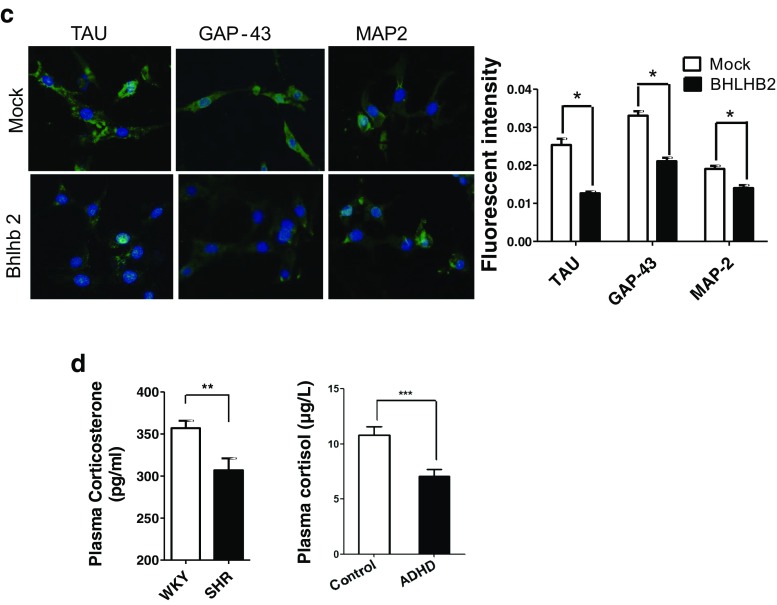
Bhlhb2 regulation neuron activity. **a** Bhlhb2 protein was detected by Western analysis in both PC12 cells and the prefrontal cortex after microinjection with interfering Bhlhb2 oligonucleotide (*top left*). Representative images of immunochemical staining of Bhlhb2 in PFC (*bottom left*). The behavior test results of SHR microinjected with small interfering Bhlhb2 oligonucleotide were summarized on the *right*. **b** The proteins of Nr3c1 and Bhlhb2 were analyzed by western (*upper*) and quantification (*right*). The neurite length or branching was analyzed (*lower*) after Bhlhb2 overexpression or target silence (siBhlhb2). Representative images of differentiated PC12 cells after incubation 10 ng/mL NGF are shown on *left* and quantitative analysis on *right. d* day, *Mock* vector control. **c** Representative micrograph of immunofluorescent staining of Bhlhb2-transfected PC12H cells with Tau, GAP-43, and MAP2 antibodies, respectively. The nucleus was stained with DAPI. **d** Plasma corticosterone and cortisol levels were measured in rats (*left*) and ADHD patients (*right*)

The common target of the miRNA, Nr3c1, and Nf1 regulation network focuses on Bhlhb2 in the PFC of SHRs. To determine the causative functions of Bhlhb2 in ADHD, we established Bhlhb2 expression silence cells and confirmed the effectiveness of the silencing oligonucleotide (Fig. 6a). The oligonucleotide modified with cholesterol was then microinjected into the PFC of seven SHRs. Results indicated that Bhlhb2 was downregulated in the PFC of these rats compared to NC injected with scramble nucleotide (Fig. 6a). The behaviors of all animals were then evaluated with the open-field and Làt maze tests within 7 days after the microinjection operation. In the open-field test, the total number of square crossings and rearings was significantly lower (Fig. 6a) in the Bhlhb2 knockdown group than in NC. In the Làt maze test, the number of corner crossings was also significantly lower in Bhlhb2 knockdown SHRs (Fig. 6a), although the number of corner rearings was not significantly different. The effects became more apparent from the second day after the microinjection operation and persisted to seventh day.

Interestingly, highly differentiated pheochromocytoma PC12H cells expressed much lower levels of Nr3c1 but higher Bhlhb2 proteins than PC12L cells. Knockdown of Bhlhb2 in PC12L cells however enhanced neurite outgrowth and branching. Overexpression of Bhlhb2 by transfection in PC12H cells resulted in significant reduction of neurite length and branching cells (Fig. 6b). The staining of axonal markers, Tau and GAP-43, was significantly suppressed in the cells with Bhlhb2 overexpression (Fig. 6c). Microtubule-associated protein 2 (MAP2), preferentially found in dendrites and neuronal somata, was also significantly reduced after Bhlhb2 overexpression.

### ADHD Is Characterized by Lower Plasma Cortisol Concentrations

Glucocorticoids work through the glucocorticoid receptor (Nr3c1), which modulates target gene transcription. Therefore, we further investigated the blood glucocorticoid in animal model of ADHD and human beings with ADHD. Our results revealed that mean plasma corticosterone concentration was significantly lower in 12 SHRs than in nine WHY rats. In the 30 boys with a diagnosis of ADHD, the plasma cortisol concentration was significantly lower than in the control group (Fig. 6d), indicating that glucocorticoid concentrations were decreased in ADHD.

## Discussion

Attention deficit hyperactivity disorder is characterized with inattention, impulsivity, and hyperactivity that affect approximately 5.3 % of children worldwide. However, the key factors with dysregulation and malfunction in the brain for the development of hyperactivity still remain unknown. Through a large scale screen, we identified a regulatory network in the PFC which is implicated in planning complex cognitive behaviors and the major affected brain area in ADHD. Based on the miRNA and cDNA microarray and proteomic data, miR-138, 138*, 296, 34c*, 494, CUX1, Pou1f1, Sp1, Nf1, and Nr3c1 were highly focused as a result of their strong connection to the differentially expressed genes and proteins in the PFC of SHR brain. The downregulated expressions of miRNA-138-2, 296, 34c*, and 494 were all confirmed in the PFC of SHR, a well-established animal model of ADHD [6, 11]. However, the expression of Nr3c1 which helps mediate executive functions, including depression and stress-related emotion [19] and biochronometer activities [20], was significantly higher in the PFC of SHRs than in WKY rats. Interestingly, the promoter regions of all miR-138, 138*, 296, 34c*, and 494 precursor genes contain the binding consensus of Nr3c1, a glucocorticoid receptor, and the ligand-activated transcription factors. Nr3c1 was elevated in the PFC, and ectopic expression of Nr3c1 indeed suppressed the promoter activity of all miR-138-1, 296, 34c*, and 494 genes, except for miR-138-2, and downregulated miR-138*, 296, 34c*, and 494 expression, not only in PC12H cells, but also in non-neuron cells, which suggests that Nr3c1 regulates the transcription of these miRNAs. miR-138 is rich in brain tissue and is encoded by either the miR-138-2 or the miR-138-1 gene, but the expression of miR-138 in the PFC was mainly from miR-138-1 gene in SHRs.

**Fig. 7:**
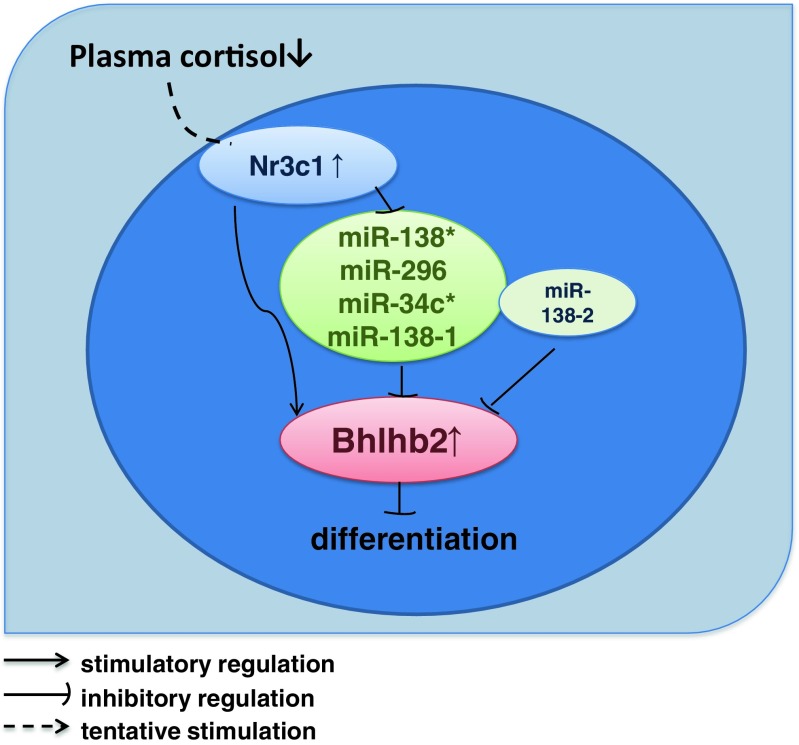
The regulation model of Nr3c1 and Bhlhb2 in ADHD. Nr3c1, miR-138, 138*, 296, 34c*, and Bhlhb2 constitute a regulatory network in the brain. Nr3c1-Bhlhb2 axis is the center of the network, and its disturbance is involved in the development of ADHD

After analysis of these miRNA targets, we surprisingly noted that all of these miRNAs have a common target Bhlhb2 mRNA. Overexpression of these five miRNAs, on the other hand, downregulated Bhlhb2 mRNA. Therefore, the abnormally high expression of Nr3c1 suppressed miRNA-138*, 296, 34c*, and 494 and enhanced Bhlhb2 expression in SHRs. Bhlhb2 is a member of the basic helix-loop-helix (BHLH) superfamily of transcription factors, an important transcription suppressor for BDNF, which links it to neuronal activity and locomotor control. In PC12 cells, we observed that Nr3c1 and Bhlhb2 were highly expressed in lowly differentiated PC12L cells. Silence of Bhlhb2 expression enhanced the neurite outgrowth and branching in PC12L, while overexpression of Bhlhb2 significantly suppressed the differentiation of PC12H cells. To further demonstrate the roles of Bhlhb2 in hyperactivity regulation, we knocked down the expression of Bhlhb2 in the PFC of SHR and interestingly observed that in vivo knockdown of Bhlhb2 in the PFC indeed significantly improved the inattention hyperactivity behaviors of SHRs, which provided the first evidence that Bhlhb2 is important in hyperactivity development. Bhlhb2 should be the common downstream key molecule of Nr3c1 and miRNA regulatory pathways responsible for the dysregulation involving attention deficit hyperactivity (Fig. 7). Nr3c1 and Bhlhb2 may constitute an important regulatory axis in the brain. Disturbance of this axis may become the mechanism involving attention deficit and hyperactivity. Abnormal expression or tyrosine phosphorylation of Nr3c1 may be the cause of the disturbance of Nr3c1-Bhlhb2 axis.

Since Nr3c1 and Bhlhb2 are also biochronometer-related [21], abnormal expression of these molecules may disturb circadian rhythms and sleep patterns, both of which are actually often found in patients with ADHD. In particular, disturbances in the circadian variations of cortisol concentrations and lower cortisol concentrations in children with ADHD, especially those with the hyperactive-impulsive type ADHD, have been documented [22]. Dysfunction in the hypothalamo-pituitary-adrenal (HPA) axis is generally considered to be responsible for low blood cortisol concentrations, which in turn might be related to the core ADHD symptoms of attention deficit, hyperactivity, and impulsive behavior [23]. Elevated expression of Nr3c1 in the PFC and hippocampus enhances the sensitivity of the response to glucocorticoid negative feedback and reduces the release of cortisol [24, 25]. Children with ADHD have a blunted cortisol response to psychosocial stressors, a decreased cortisol awakening response, and lower plasma daytime cortisol concentrations. Family-based association tests indicate that Nr3c1 single-nucleotide polymorphism is associated with HPA axis reactivity [26] and ADHD morbidity rate [27]. The disturbance in the HPA axis in ADHD has been thought to be related with an excessive exposure to glucocorticoids in the fetal and early postnatal periods. Current data indicate that higher levels of Nr3c1 in the PFC and hippocampus strengthen negative feedback regulation of the HPA axis. Whether excessive exposure to glucocorticoids in early postnatal period will cause overexpression of Nr3c1 in the PFC needs future investigation.

## Electronic supplementary material

Below is the link to the electronic supplementary material.

ESM 1 (PDF 384 kb)
