# Supplementary material for: Nr3C1-Bhlhb2 Axis Dysregulation Is Involved in the Development of Attention Deficit Hyperactivity
Source: Mol Neurobiol. 2016 Jan 28;54(2):1196–212. doi: 10.1007/s12035-015-9679-z (PMC5310568; doi:10.1007/s12035-015-9679-z)
Supplement: Supplementary file 1 — (PDF 384 kb) [file 12035_2015_9679_MOESM1_ESM.pdf]

Supplemental Table 1.

Primer Sequences used in this study for PCR or cloning assays.

Gene	Primer (5→3)
	**qPCR**
CUX1	F: TGTCTCCACCTCCTTCCA
	R: GCAGTGCAGTGAGACCCT
POU1F1	F: ATGCCGCTGAGGGTCT
	R: TTTCCGCCTGAGTTCC
Nr3c1	F: ATGGGCAAAGGCGATA
	R: GAGATACTCTTCATAGGATACCT
Nf1	F: AGGAGACGCTGAAGGAGT
	R: GTACCAGGATCGGTGAGG
Sp1	F: ATCTCACTTCCCAAACTCAT
	R: TTGTGCTCCTCAATCTCC
BDNF	F: TTATTTCATACTTCGGTTGC
	R: ACCTTCTGGTCCTCATCC
Bhlhb2	F: AACTCTTGAGGGCGAATC
	R: AGGCCATCCTTCTCACAT
GAPDH	F: CGTATCGGACGCCTGGTTA
	R: CGCTCCTGGAAGATGGTGA
Pri-miR-138-1	F:5’-TGTCTCCACCTCCTTCCA-3’
	R: 5’-GCAGTGCAGTGAGACCCT-3’
Pri-miR-138-2	F:5’-CTTTCTAACCGACTGGAGC-3’
	R: 5’-ACCCTGGTGTCGTGAAA-3’
Pri-miR-296	5’-CACCACCTTCCCATTGTC-3’
	R: 5’-AGAGCCTCCACCCAACC-3’
Pri-miR-34c*	F:5’-AAAAGGATGCCAGGAAGA-3’
	R: 5’-GTCTTTTTACCTGGCTGTGT-3’
Pri-miR-494	F:5’-GCTGCCTTTGTTTGCTTT-3’
	R: 5’-ACTAAAAAAGAGGTTTCCCG-3
mir-138	RT:GTCGTATCCAGTGCAGGGTCCGAGGTATTCGCACTGGATACGACCGGCCTG
	F:gctgcAGCTGGTGTTGTGA
	R:GTGCAGGGTCCGAGGT
mir-138*	TR:GTCGTATCCAGTGCAGGGTCCGAGGTATTCGCACTGGATACGACCCCTGG
	F:Gagaacggctacttcacaa
	R:GTGCAGGGTCCGAGGT
mir-296	RT:GTCGTATCCAGTGCAGGGTCCGAGGTATTCGCACTGGATACGACGGAGAG
	F:cttcaGAGGGTTGGGTGGA
	R:GTGCAGGGTCCGAGGT
mir-34c*	RT:GTCGTATCCAGTGCAGGGTCCGAGGTATTCGCACTGGATACGACCCTGGC

	F:gtaccAATCACTAACCACA
	R:GTGCAGGGTCCGAGGT
mir-494	RT:GTCGTATCCAGTGCAGGGTCCGAGGTATTCGCACTGGATACGACAGAGGT
	F:Tatgatgaaacatacacgg
	R:GTGCAGGGTCCGAGGT
RNU6	RT: GCTTCGGCAGCACATATACT
	F: AACGCTTCACGAATTTGC
	R: AAAATATGGAACGCT

Cloning

miR-138-1 promoter	F:CCAGGTTCAAATCCAAG
	R:AGGGCAGATAGTTGATGAT
miR-138-2 promoter	F: TGGGGAACAGGAGTCAAG
	R: ACCATACCGGAATTAGCG
miR-494 promoter	F: CAGCCAGCATTACCTCAT
	R: CCCGTTACAATTCGACAG
miR-296 promoter	F: TAGGTCCCTTTTTGTGGC
	R: TCCTTTCTTGTCATCATCC
miR-34c* promoter	F: TCACTCCTTCCTCCCTAAC
	R: CTCTTCCTGGCATCCTTT
Bhlhb2 promoter	F: 5’-ACAGAAGCAAATAGCGATGGAA-3’
	R: 5’-GGAGGGAAAGGGGGGGT-3’
Bhlhb2 3’UTR	F: 5’-AACTCTTGAGGGCGAATCTC-3’
	R: 5’-ACGAGGGGGCATCTCTGAG-3’
Bhlhb2 CDS	F: AAAGAATTCATGGAGCGGATCCCC
	R: GGGTCTAGAGTTTAGTCTTTGGTTTCTAAGTTT’
Nr3c1 CDS	F: 5’-AATGGACTCCAAAGAATCCT-3’
outside	R: 5’-CATGCCTCCACGTAACTGT-3’
inside	F: 5’-ATGGACTCCAAAGA-3’
	R: 5’-TTTTTGATGAAACA-3’
CUX CDS	F:ATGGAGCGCGCCTCAGGTC
	R:CCCTCAGAACTCCCATTCAATGG
BDNF CDS	F:ACCAGGTGAGAAGAGTGA
	R:TCCACTATCTTCCCCTT
miR-138-1 pre	F: AGCAGCACGCAGAGCAG
	R: GAAGGAAACAAAGGGGGAA
miR138-2 pre	F: GCTGCTGTAGACCTGATA
	R:AGCCGTTGAGTGATGTAA
mir-296 pre	F: CCAACAGTTTCCTTCATT
	R:AGATCAGTGGCAGCTTAC
mir-34c pre	F:CAAGTAACCCTCGGAGACC
	R:TTTTGGAGAAGGAAATGGAC
mir-494 pre	F:ATCATTCCTGAACACCCAC
	R:AGGTATCATCCCACAGACG

Supplemental Table 2. Predicted Binding Sites for the Transcription Factors

Mature miRNAs	miRNA genes	Chr (strand)	Host gene	Predicted CUX1 or NR3C1 or POU1F1 or SP1 or NF1 binding sites
miR-138	pre-mir-138-2	8(+)	none	NF-1/L :TGGCA (-182 to -186) ,(-545 to -549), (-527 to -532), (-527 to -532),(-1601 to -1605),(-1535 to -1539), (-1017 to -1021)<br>Pit-1a : TAAAT (-876 to -880) (-863 to-867) (-859 to -863) (-681- -687) (- 678 to-687) (- 418 to -422) (-1122 to-1126) (-1328 to-1334) (-1151 to -1155) (-59 to-63) (-1011 to-1017) (-2221 to -2225) (-2287 to -2291) (-2460 to -2466)<br>CDP2：CCAAT (-47 to -51), (-603 to- 607)<br>SP1: TGTGC (-345 to -349) (-1766 to 1770) (- 1478 to - 1482) (-1012 to – 1016) (- 2045 to -2050) (-1179 to - 1187) (- 208 to – 212)<br>GR: CAGAG (- 975 to - 979) (- 1697 to -1701) (- 1402 to – 1406) (- 524 to – 528) (- 1833 to – 1837) (- 501 to – 505) AGAACA (- 481 to – 486) (- 673 to – 678) (- 217 to -222) (-1457 to – 1462)CACCC (- 228 to -232) (- 494 to – 498) (-596 to – 600) (- 1414 to – 1418) GACACA (- 1907 to – 1912) TGAACT (- 9 to – 14) TGTGCC (- 345 to – 350) TATAT (-679 to – 683) (- 1128 to – 1132) (- 2024 to -2028) TGTCCT (- 2070 to – 2075)
miR-138*	pre-mir-138-1	19(-)	none	NF-1/L :TGGCA (- 528 to – 532) (- 1601 to – 1605) (- 1535 to – 1539) (- 1017 to – 1021)<br>Pit-1a : TAAAT (- 1122 to – 1126) (- 1328 to – 1334) (- 1650 to – 1654) (- 59 to – 63) (- 2287 to – 2291) (- 2460 to – 2466)<br>CDP2：CCAAT (- 603 to – 607) (- 1540 to – 1547)<br>SP1: TGTGC (- 208 to – 212) AAGGCTGGA (- 1179 to – 1187)<br>GR: TGTCC (- 766 to – 771) TGAACT (- 613 to – 618) (- 142 to – 146) CACCC (- 248 to – 252) (- - 971 to – 975) (-354 to – 358) TGTGAT (- 1674 to – 1679) TGTTCT (- 853 to 858) (-2246 to – 2251) (- 2368 to – 2373) (- 1461 to – 1466) TGTGCC (- 2329 to 2334) CAGAG (- 977 to – 981) (- 11 to – 15) (- 1328 to 1334) (- 2385 to – 2389)
	pre-mir-138-2	8(+)	None	NF-1/L :TGGCA (- 1404 to – 1412) (- 182 to – 186)<br>CDP2: CCAAT (- 794 to – 798)<br>Pit-1a: TAAAT (- 876 to – 880) (- 863 to – 867) (- 859 to – 863) (- 681 to – 687) (- 418 to – 422)<br>SP1: TGTGC (- 345 to – 349) (--1766 to – 1770) (- 1478 to -1482) (- 1012 to – 1016) CCGCCC (- 2045 to – 2050)<br>GR: CAGAG (-501 to – 505) (- 1697 to – 1701) (- 9 to – 14) (- 1402 to – 1406) (- 2238 to – 2242) (- 524 to – 528 ((- 975 to – 979) (- 1833- to - 1837) (- 53 to – 57) TATAT (- 2024 to – 2028) (- 679 to – 683) (- 1128 to – 1132) AGAACA (- 217 to – 222) (- - 673 to - 678) (- 1457 to – 1462) (- 481 to – 486) TGTCCT(- 2070 to – 2075) CACCC (- 228 to – 238) (- 1414 to – 1418) (- 494 to – 498) (- 596 to – 600) GACACA (- 1907 to – 1912) TGTGAT (- 1074 to – 1079) TGTGCC (- 345 to – 350)

miR-296	pre-mir-296	3(-)	none	NF-1/L :TGGCA (-578 to 582) (- 1213 to – 1217) (- 1212 to – 1217)<br>Pit-1a : TAAAT (- 342 to – 346) (- 2382 to – 2388)<br>CDP2：CCAAT (- 1199 to – 1203) (- 1667 to – 1671)<br>SP1: TGTGC (- 930 to – 934) (- 710 to – 714) (- 702 to – 706) (- 688 to – 692) (- 679 to – 683) (- 1590 to – 1594) (- 2467 to – 2471)<br>(- 2191 to – 2195) (- 1786 to – 1790)<br>GR: TGAACT (- 502 to – 507) (- 1272 to – 1277) CAGAG (-131-to 135) (- 613 to – 617) TGTGCC (- 710 to – 715) (- 1365 to – 1370) GACACA (- 836 to 841) (- 227 to – 232) TGTCCT (- 1555 to – 1560) (- 1725 to – 1730) (- 2174 to – 2179)) (- 2428 to – 2433) TGTGAT (- 1573 to – 1578) TATA (- 371 to – 375) TGTCCC (- 2056 to – 2061) CACCC (- 1748 to – 1752) (- 1883 to – 1887) (- 939 to – 943) (- 1877 to – 1881) (- 1463 to – 1467) AGAACA (- 2447 to – 2452) (- 73 to – 78) TGTTCACC (- 1744 to – 1751)
miR-34c*	pre-mir-34c	8(-)	None	NF-1/L :TGGCA (- 2458 to – 2462) (- 2062 to – 2066) (- 1492 to – 1497) (- 157 to – 161) (- 281 to – 285) (- 1493 to – 1497) (- 2029 to – 2033) (2061 to – 2066)<br>Pit-1a : TAAAT (- 417 to – 423) (- 2495 to – 2499) (- 524 to – 530)<br>CDP2：CCAAT (- 2351 to – 2355)<br>SP1: TGTGC (- 136 to – 140) (- 1301 to – 1305) (- 1972 to – 1976) (- 2168 to – 2172) (- 2320 to -2324) (- 2443 to 2447)<br>GR:AGAACA (-1143 to – 1148) CACCC (- 1496 to – 1500) (- 1104 to – 1108)TGTCCT (- 1253 to – 1258) (- 973 to – 978) CAGAG (- 644 to – 648) (- 638 to – 642) (- 2432 to – 2436) (- 2254 to – 2258) (- 1221 to – 1225) GACACA (- 2117 to – 2122) (- 1721 to – 1726) (- 1592 to – 1597)TATAT (- 2452 to – 2456) (- 2162 to – 2166) TGTGCC (- 2443 to – 2448) (- 2320 to – 2325) (- 2168 to – 2173) TGTCCC(- 1804 to – 1809)
miR-494	pre-mir-494	6(+)	none	NF-1/L :TGGCA (- 243 to – 247) (- 1155 to – 1159) (- 1354 to 1358) (- 2039 to – 2043) (- 1742 to – 1748)<br>Pit-1a : TAAAT (- 124 to – 130) (- 413 to – 419) (- 877 to – 881) (- 958 to – 962) (- 2109 to – 2113)<br>CDP2：CCAAT (- 1744 to – 1748) (- 1068 to – 1072)<br>SP1: TGTGC (- 1881 to – 1885) (- 1007 to – 1011)<br>GR: AGAACA (- 788 to – 793) CAGAG (- 2190 to – 2194) (- 585 to 589) (- 132 to – 136) TATAT (- 2069 to – 2073) (- 1962 to – 1966) (- 1301 to – 1305) (- 952 to – 956) (- 446 to – 450) TGTGCC (- 1881 to – 1886) TGTTCT (- 1109 to – 1114) (- 1784 to – 1789) GACACA(- 1025 to – 1030) TGCCT （ - 854 to – 859) TGAACT (- 861 to – 866)
Bhlhb2		4(+)		NF-1/L: TTGGCA(-65 to -70), (-1139 to 1144),(-1513 to -1518) TGGCA(-66 to 70),(-1140 to -1144),(-1514 to -1518),(-1520 to 1524),(-2139 to -2143),(-2423 to -2427)<br>Pit-1a: TAAAT(-1121 to -1125),(-1183 to -1187),(-1781 to -1785),(-2212 to -2216)<br>AATTCAG(-1128 to -1134)<br>CDP2: CCAAT(-510 to -514),(-680 to -684),(-1967 to -1971)<br>Sp1: TGTGC(-1374 to -1378),(-1605 to -1609),(-1685 to -1689),(-1973 to -1977),(-2072 to -2076)<br>GR: AGAACA(-1845 to -1850),(-1873 to -1878)<br>CACCC (-1380 to -1384),(-1536 to -1540)<br>CAGAG(-283 to -287),(-861 to -865),(-2224 to -2228),(-2255

				to -2259)<br>TATAT(-1938 to -1942),(-1940 to-1944) TGTCCC(-125 to -130) TGTGAT(-577 to -582) TGTGCC(-1685 to -1690) TGTTCT(-1255 to -1260)
